# Evaluation of deep-learning iterative reconstruction combined with high-frequency kernels in CT: a task-based image quality study

**DOI:** 10.1186/s41747-026-00801-3

**Published:** 2026-09-10

**Authors:** Anaïs Viry, Pascal Monnin, Chiara Pozzessere, Damien Racine

**Affiliations:** 1 Institute of Radiation Physics (IRA), Lausanne University Hospital and University of Lausanne, Lausanne, Switzerland; 2 Department of Radiology and Interventional Radiology, Lausanne University Hospital and University of Lausanne, Lausanne, Switzerland

**Keywords:** Bone and bones, Deep-learning, Thorax, Tomography (x-ray computed)

## Abstract

**Objective:**

To evaluate the image quality of a novel deep-learning iterative reconstruction (DLIR) algorithm combined with high-frequency kernels compared with iterative reconstruction (IR) algorithms through the assessment of the detectability of small high-contrast lesions.

**Materials and methods:**

Three image-quality phantoms were scanned with doses from 0.8 to 15 mGy. Images were reconstructed using ASIR-V0, ASIR-V80, and DLIR (Low, Medium, High), with an edge-enhancing kernel for a chest protocol and a sharp kernel for a spine protocol. A dedicated cubic phantom was used to assess axial, longitudinal resolution, and noise power spectra. Contrast of calcium-based lesions of 3 and 5 mm in diameter was assessed at three concentrations (200, 400, and 800 mg/cc) using two anthropomorphic chest and abdominal phantoms. Detectability was evaluated using a non-prewhitening with eye filter model observer.

**Results:**

In-plane spatial resolution was stable across DLIR strength levels and slightly better than ASIR-V0, while longitudinal resolution did not depend on algorithms. DLIR markedly reduced image noise, below ASIR-V80 for DLIR-High with both kernels. While IR preserved the contrast of lesions, it decreased with DLIR strength for the small lesion 200 mg/cc-3mm. The highest detectability was achieved with DLIR-High, except for the HA200-3mm below 3 mGy for the chest protocol and 7 mGy for the spine protocol.

**Conclusion:**

DLIR combined with high-frequency kernels reduced image noise while preserving spatial resolution and FBP-like noise texture, outperforming IR in detectability for most lesion sizes, concentrations, and dose levels. DLIR reduced contrast for small and low-concentration lesions, especially at low doses, which partially counterbalanced its detectability advantage over IR.

**Key Points:**

- ***Question*** This study evaluates whether deep-learning reconstruction with high-frequency kernels improves the detectability of small high-contrast lesions compared to iterative reconstruction.
- ***Findings*** The new deep-learning reconstruction reduces image noise while preserving fine anatomical detail and improving in-plane spatial resolution.

***Relevance statement*:**

The new deep-learning reconstruction combined with high-frequency kernels improves detection of small lesions in comparison with iterative reconstruction at standard doses, but very small or low-density lesions may appear less visible at lower dose levels.

**Graphical Abstract:**

**Figure:**
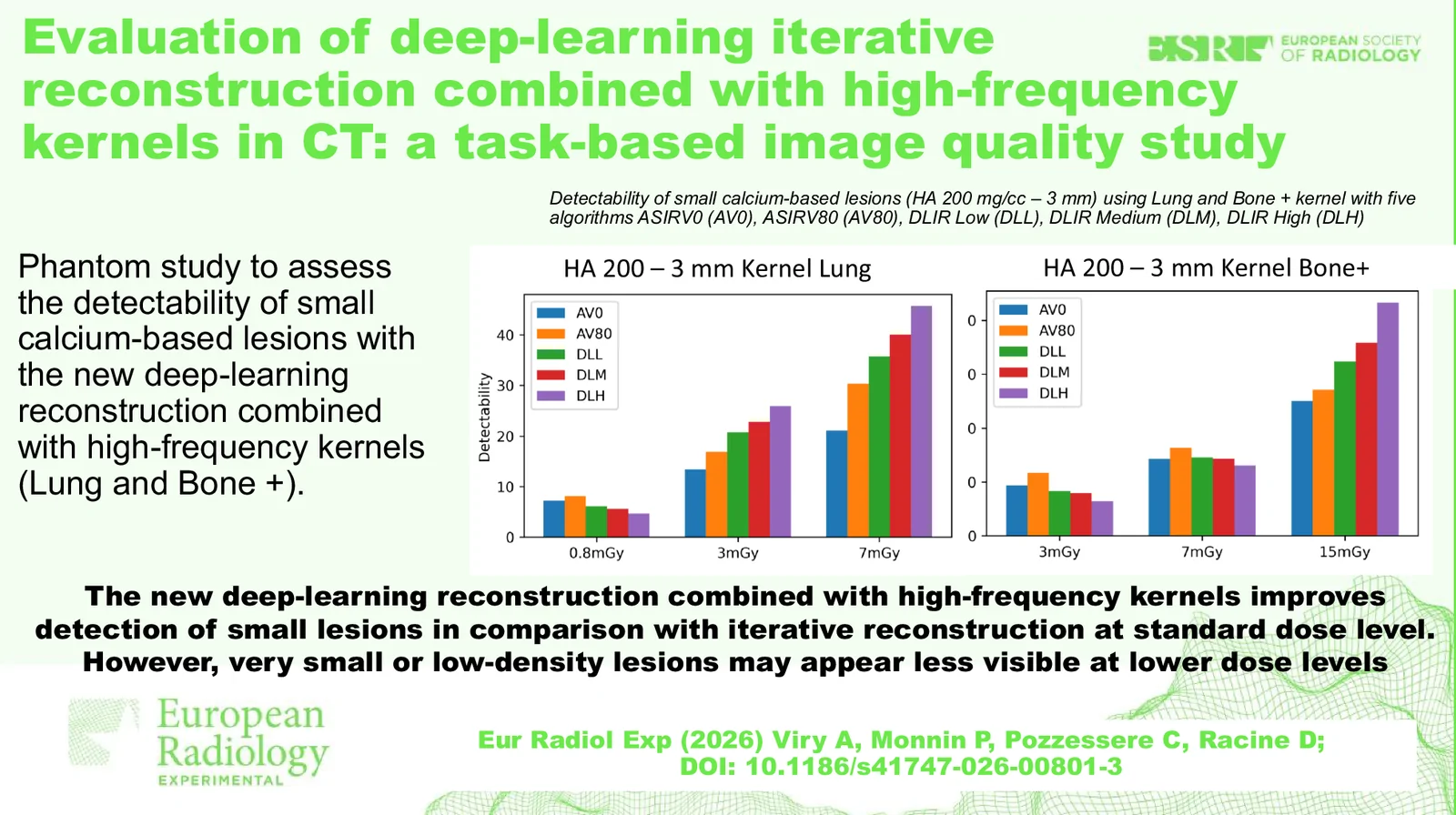

## Background

Over the past decade, iterative reconstruction (IR) algorithms have been widely implemented in computed tomography (CT) to reduce image noise while preserving spatial resolution and improving diagnostic image quality at lower patient radiation exposure [1–5]. In comparison with filtered back projection (FBP), IR enables substantial dose reduction for various protocols [6–8]. However, IR algorithms are often associated with non-linear noise behavior and artificial image textures, which may affect image appearance and diagnostic confidence, particularly at high strengths [9, 10].

More recently, deep-learning–based reconstruction algorithms have been introduced to further reduce noise while maintaining a more natural image texture [11, 12]. In 2020, the deep-learning iterative reconstruction (DLIR) algorithm was introduced and was initially available only for soft-tissue kernels. This algorithm relies on a convolutional neural network trained with low-noise and high-quality images, enabling the model to selectively reduce noise while preserving anatomical details [13]. Several phantom and clinical studies have demonstrated superior image quality and significant dose reduction across multiple clinical contexts [14–18]. In chest imaging, the added value of DLIR has been shown in nodule-related tasks, showing performance that is comparable or superior to IR, while significantly reducing radiation exposure [19]. High-frequency reconstruction kernels are routinely used in clinical CT protocols requiring high spatial resolution, such as chest parenchyma imaging or musculoskeletal applications. Unlike smooth kernels, they amplify high-frequency spatial content to improve edge definition and resolve fine structures such as small vessels, bronchovascular bundles, or trabecular bone, at the cost of markedly increased image noise and a grainier appearance. In high-noise conditions, the application of denoising algorithms is both particularly relevant and challenging, as it requires effectively distinguishing signal from noise, especially in the case of small lesions with low contrast. More recently, GE Healthcare extended its DLIR algorithm to high-frequency kernels, making it available for the first time for these high-resolution protocols. Kawai et al [20] demonstrated improved delineation of pancreatic cancer margins in clinical images using this high-frequency kernel. However, to our knowledge, no objective task-based image quality study has yet evaluated this new combination.

This study aimed to perform the first phantom task-based characterization of DLIR combined with high-frequency kernels by evaluating noise reduction, spatial resolution, contrast, and lesion detectability of small high-contrast lesions across a range of dose levels, in comparison with iterative reconstruction algorithms.

## Materials and methods

### Phantoms acquisition

**Fig. 1:**
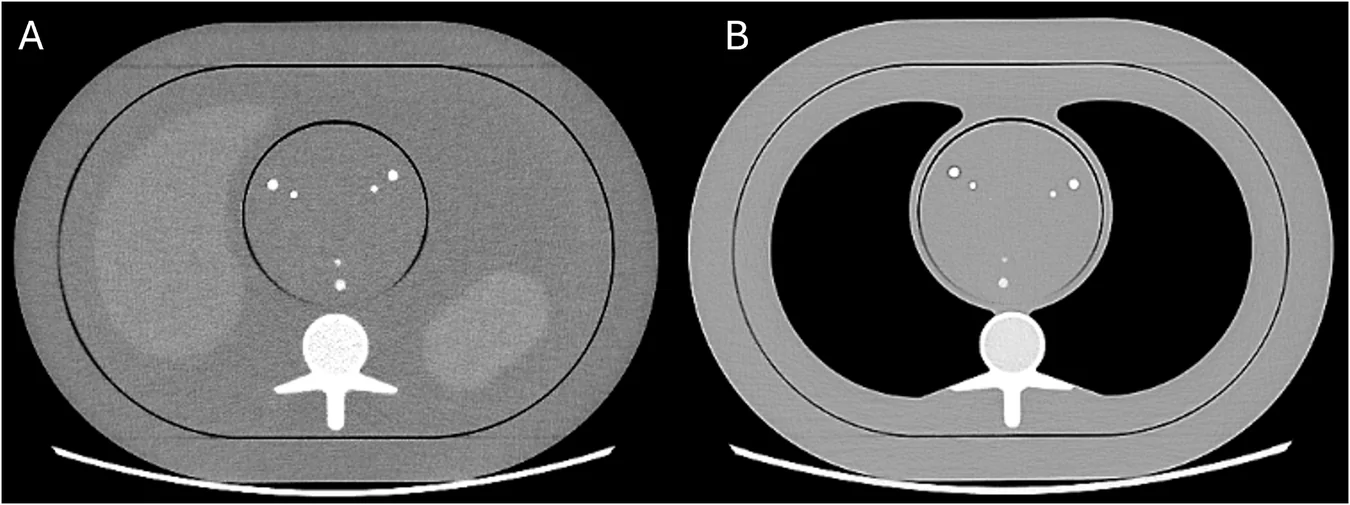
CT image of the small calcium-based lesions included in the abdominal phantom (**A**) and the chest phantom (**B**)

Three dedicated phantoms were used to assess task-based image quality. Firstly, a water-filled cubic test object (160 mm side, rounded corners to avoid streak artifacts) with a removable central polymethyl methacrylate (PMMA) rod (70 mm diameter and height) was used to measure in-plane spatial resolution, longitudinal spatial resolution, and noise power spectra. The rod was positioned successively along the z-direction (head-foot) and along the y-direction (antero-posterior) to assess in-plane and longitudinal resolution, respectively. The PMMA/water interface produced a contrast of 120 HU at 120 kVp. Noise was measured in the homogeneous water volume without the central rod. Second, two elliptic semi-anthropomorphic thoracic and abdominal phantoms (QRM, A PTW COMPANY, Möhrendorf, Germany) were used to assess the contrast of calcium-based lesions for the chest and lumbar spine protocols, respectively. A dedicated phantom module, inserted successively in both phantoms, contained nine cylindrical hydroxyapatite calcifications embedded in a soft-tissue-equivalent material. The module included three diameters (1, 3, and 5 mm) and three concentrations (200, 400, and 800 mg/cc), resulting in a total of nine cylinders, each with a height equal to its diameter (Fig. 1).

Each phantom was scanned on a GE Revolution Apex Elite (GE Healthcare, Milwaukee, MI) with fixed parameters: 120 kVp, pitch 0.992, rotation time 0.35 s, collimation 64 × 0.625 mm (total beam width: 40 mm), slice thickness 1.2 mm, increment 0.625 mm. The edge-enhancing “Lung” kernel was used for the chest protocol, and the high-resolution “Bone+” kernel was chosen for the lumbar spine protocol. The tube current was varied to achieve a range of dose levels from low-dose to standard clinical dose. The cubic phantom was scanned at 0.8, 3.0, and 7.0 mGy (60, 115, 270 mA) for the chest protocol and at 3.0, 7.0, and 15.0 mGy (115, 270, 575 mA) for the lumbar spine protocol five times per dose level. The abdominal phantom (lumbar spine protocol) was scanned at 0.5, 0.8, 1.5, 3.0, 7.0 and 15.0 mGy (20, 30, 60, 115, 270, 575 mA), and the thoracic phantom (chest protocol) at 0.2, 0.5, 0.8, 1.5, 3.0 and 7.0 mGy (10, 20, 30, 60, 115, 270 mA) ten times per dose level. The displayed field of view was adjusted to encompass the phantoms, set at 200 mm for the cubic phantom and at 370 mm for the anthropomorphic phantoms. All the images were reconstructed using an IR algorithm at two levels, ASIR-V0 (AV0) and ASIR-V80 (AV80), and DLIR at three levels: Low (DLL), Medium (DLM), and High (DLH).

### Task-based image quality metrics

The detectability of small calcium-based lesions was evaluated using a mathematical observer that incorporates in-plane and longitudinal spatial resolution, noise, and lesion contrast measured in phantom images.

#### In-plane spatial resolution

Axial images of the cubic phantom with the PMMA rod aligned along the z-direction were used to assess the in-plane target transfer function (TTF) using the circular edge between the PMMA rod and the surrounding water [21, 22]. Regions of interest (ROIs) of 100 × 100 mm^2^ centered in the PMMA rod were extracted from 80 CT slices. To secure a reliable estimate of the TTF (calculation uncertainty below 10%), Chen et al recommend a minimum contrast-to-noise ratio (CNR) equal to 15 [23]. By averaging the 80 CT slices, the CNR of the PMMA edge was equal to 57 in the most unfavorable condition (lowest CTDIvol, AV0, kernel “Lung”). HU value profiles perpendicular to the PMMA–water interface, computed over 10° angular apertures, were used to derive radial edge spread functions. The data were analyzed in 5° increments, yielding 72 radial edge spread functions, which were subsequently linearly resampled and Fourier-transformed to obtain pre-sampling radial TTFs. The resulting in-plane TTF is the average of the radial TTFs normalized to 1.0 at the zero-frequency.

#### Longitudinal spatial resolution

Axial images of the cubic phantom positioned with the PMMA rod perpendicular to the z-direction were used to assess the longitudinal TTF. The orthogonal xz-plane of the primary axial reconstruction was generated using independent software (Image J) without interpolation. Radial TTFs were obtained using the methodology described for in-plane resolution, and a 2D TTF was reconstructed using the angular interpolation method described by Monnin et al [24]. The TTFs along the z axis were obtained by integration of the 2D TTFs over the frequency bandwidth f_z_ using Eq. 1.

1 $${TT}{F}_{z}\left({f}_{z}\right)=\frac{\int {TT}{F}_{{xz}}\left({f}_{x},{f}_{z}\right)d{f}_{x}}{{\left.\int {TT}{F}_{{xz}}\left({f}_{x},{f}_{z}\right)d{f}_{x}\right|}_{{f}_{z}=0}}$$

This methodology was designed to obtain the in-plane and longitudinal spatial resolution using only one phantom.

#### Noise power spectrum (NPS)

The three-dimensional NPS was computed using a volume of interest of 100 × 100 × 100 mm^3^ positioned within the homogeneous water region at the center of the cubic phantom. The 3D NPS was defined by Eq. 2:

2 $${NPS}\left({f}_{x},{f}_{y},{f}_{z}\right)=\frac{\Delta x\cdot \Delta y\cdot \Delta z}{{N}_{x}\cdot {N}_{y}\cdot {N}_{z}}\cdot \frac{1}{{N}_{{VOI}}}{\left|\sum _{1}^{{N}_{{VOI}}}{FFT}\left({{VOI}}_{i}\left(x,y,z\right)-V{\bar{O}}I\right)\right|}^{2}$$

where ${{VOI}}_{i}\left(x,y,z\right)$ denotes the voxel value at location (x,y,z), and ${V}{\bar{O}}{I}$ represents the mean voxel value within the volume of interest. The parameters *N*_*x*_, *N*_*y*_, *N*_*z*_ correspond to the number of voxels, while$\,\varDelta x,\,\varDelta y,\,\varDelta z$ indicate the voxel spacing along the x, y, and z axes, respectively. The 3D NPS is expressed in HU²·mm³. No windowing or detrending was applied to the uniform images prior to NPS computation. 2D NPS were generated by integrating the 3D NPS along the frequency direction perpendicular to the selected 2D plane. One-dimensional NPS curves were obtained by angular averaging the 2D NPS over 360°, excluding the zero spatial frequency axes. Both 2D and 1D NPS are reported in HU²·mm². The noise magnitude, equal to the square root of the integral of the NPS and expressed in HU, was used to calculate the noise magnitude ratio between AV0 and the other algorithms (AV80, DLL, DLM, DLH). The average spatial frequency (f_av_, in mm^-1^) of the 1D NPS was also measured to describe noise texture. Using this high number of pixels, the uncertainties associated with the NPS calculation can be estimated to be below 10% [23].

#### Contrast

The contrast of calcium-based lesions (diameters 3 and 5 mm) was assessed in the slice containing the highest lesion signal. The mean lesion HU value S was measured within a small ROI of 3–5 pixels centered on each lesion, averaged over the ten acquisitions. A Python script was developed to automatically position each ROI within the lesion. A threshold was first applied to approximately detect the center of each lesion. ROI were then shifted around this center to identify the position yielding the maximal signal. The mean background value Sₙ was obtained from three homogeneous ROIs of 30 pixels placed around the lesion on the same slice. Contrast was defined as$\,{C}_{{HU}}=S-{S}_{n}$. Standard deviations were assessed using the ten acquisitions. The 1 mm lesion was excluded from analysis as it was below the detection threshold in all conditions.

#### Non-prewhitening model observer with eye filter (NPWE)

The detectability of small cylinders of 3 and 5 mm in height and diameter was evaluated using the mathematical observer NPWE. A good agreement with human observers’ performances has been found in previous studies [25]. This model used the measured 3D NPS, in-plane and longitudinal TTFs, and the contrast of small calcium-based lesions measured on phantom images [25]. The detectability index was computed by assuming the separability between the in-plane (TTF_xy_) and longitudinal TTF (TTF_z_) using Eq. 3. A home-made software developed in IgorPro version 10.02 (WaveMetrics, Inc., Lake Oswego, OR, USA) was used to perform the calculations.

3 $$d^{\prime} =\frac{{C}_{HU}\cdot {\int }_{-{f}_{x,Nyq}}^{{f}_{x,Nyq}}{\int }_{-{f}_{y,Nyq}}^{{f}_{y,Nyq}}VT{F}^{2}({f}_{x},{f}_{y})\cdot TT{F}_{xy}^{2}({f}_{x},{f}_{y})\cdot {\left({\int }_{-{f}_{z,Nyq}}^{{f}_{z,Nyq}}TT{F}_{z}({f}_{z}){S}_{obj}({f}_{x},{f}_{y},{f}_{z})d{f}_{z}\right)}^{2}d{f}_{x}d{f}_{y}}{\sqrt{{\int }_{-{f}_{x,Nyq}}^{{f}_{x,Nyq}}{\int }_{-{f}_{y,Nyq}}^{{f}_{y,Nyq}}VT{F}^{4}({f}_{x},{f}_{y})\cdot TT{F}_{xy}^{2}({f}_{x},{f}_{y})\cdot \left({\int }_{-{f}_{z,Nyq}}^{{f}_{z,Nyq}}NP{S}_{HU}({f}_{x},{f}_{y},{f}_{z})\,d{f}_{z}\,\right)\cdot {\left({\int }_{-{f}_{z,Nyq}}^{{f}_{z,Nyq}}TT{F}_{z}({f}_{z}){S}_{obj}({f}_{x},{f}_{y},{f}_{z})d{f}_{z}\right)}^{2}d{f}_{x}d{f}_{y}}}$$

The Fourier spectrum ${S}_{{obj}}\left({f}_{x},{f}_{y},{f}_{z}\right)$ of a rod of radius *R* and height *2R* is given in Eq. 4, where J_1_ is the Bessel function of the first kind of the first order.

4 $${S}_{{obj}}\left({f}_{x},{f}_{y},{f}_{z}\right)=R/f\cdot {J}_{1}\left(2\pi {Rf}\right)\cdot {sinc}\left(2\pi R{f}_{z}\right)$$

where $f=\sqrt{{{f}_{x}}^{2}+{{f}_{y}}^{2}}$. The visual transfer function (VTF) with a general formulation given in Eq. 5 was used to take into account the contrast sensitivity of the human eye [26, 27].

5 $${VTF}\left({f}_{x},{f}_{y}\right)={f}^{n}\cdot exp \left(-c{f}^{a}\right)$$

The parameters *n*, *c*, and *a*, equal to 1.0, 2.2 mm^-1^ and 1.0, respectively, were originally described by Gang et al in 2014 for CT applications [25]. Because the NPWE detectability index is derived from the NPS, the TTF, and the task function rather than being measured directly, and as no validated method for estimating its uncertainty is currently available, no standard deviation was computed for the model-observer results. Results are reported descriptively only.

## Results

### In-plane and longitudinal spatial resolution

**Fig. 2:**
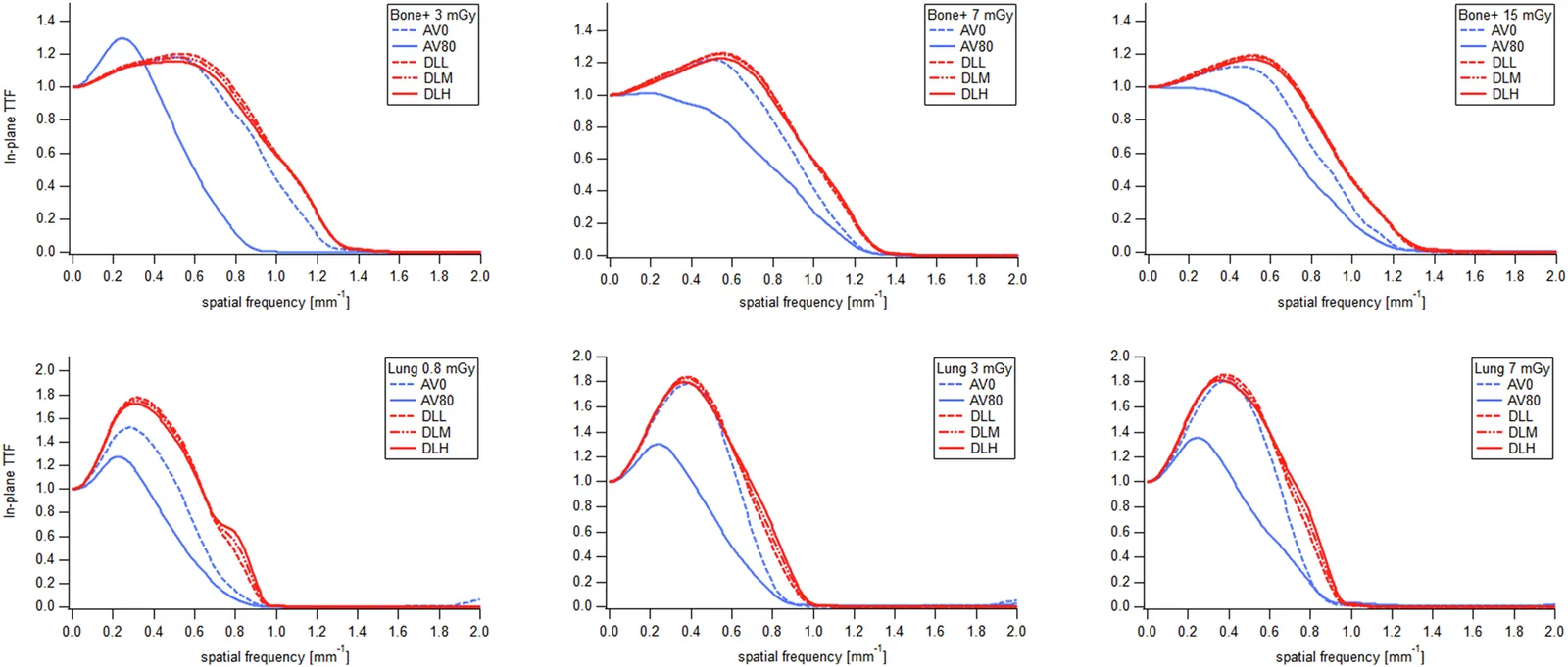
In-plane spatial resolution described by the in-plane TTF for the lumbar spine protocol (reconstructed with the kernel Bone+) and the chest protocol (reconstructed with the kernel Lung) at three dose levels with five different algorithms, ASIR-V0 (AV0), ASIR-V80 (AV80), DLIR-Low (DLL), DLIR-Medium (DLM), and DLIR-High (DLH)

**Fig. 3:**
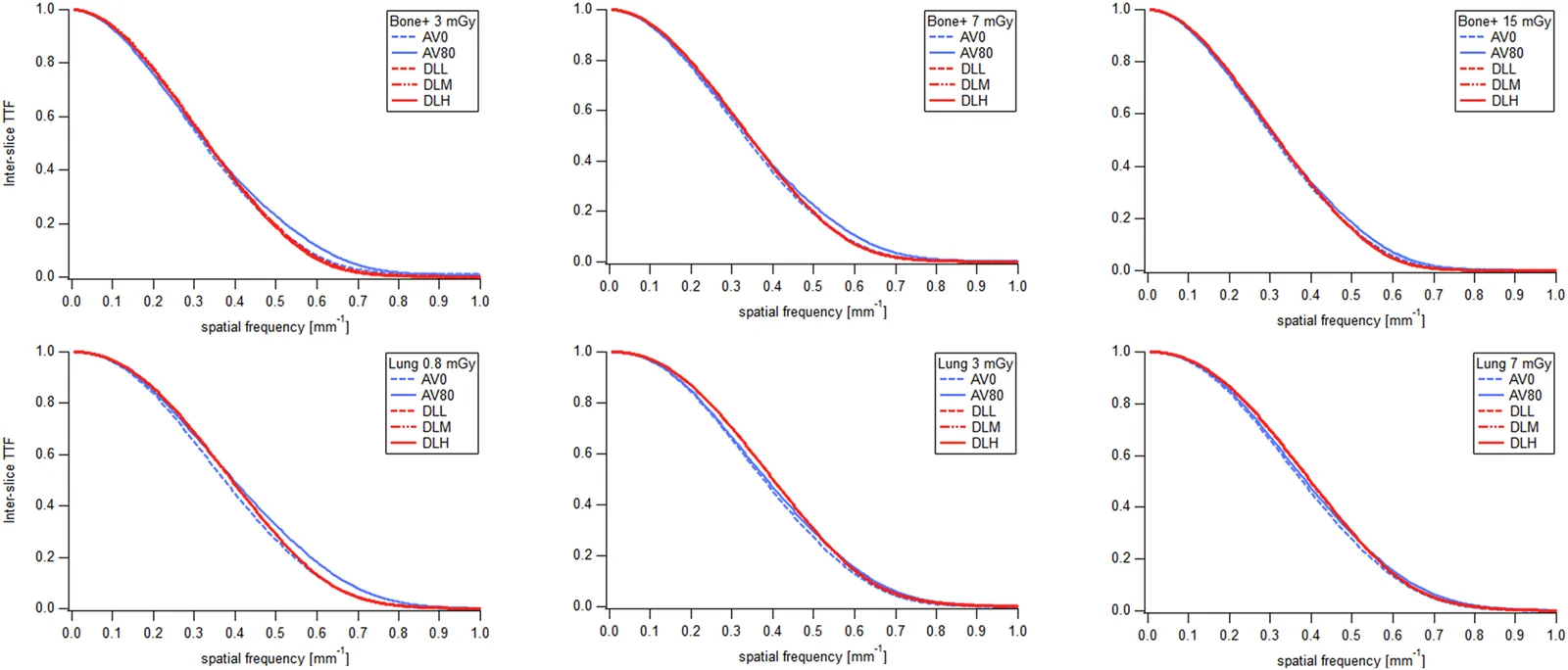
Longitudinal spatial resolution (TTF_z_) for different dose levels/kernels for the five algorithms ASIR-V0 (AV0), ASIR-V80 (AV80), DLIR-Low (DLL), DLIR-Medium (DLM), and DLIR-High (DLH)

The in-plane TTF demonstrated for both kernels and all dose levels an edge enhancement, which was more pronounced for the Lung kernel (up to 1.8) than for the Bone+ kernel (up to 1.3) (Fig. 2). The in-plane TTFs were very similar across DLIR strengths (Low, Medium, High), regardless of dose level and kernel. In contrast, differences were observed between ASIR-V strengths. ASIR-V80 exhibited markedly lower spatial resolution compared with ASIR-V0 and all DLIR strengths. The peak frequency for ASIR-V80 shifted toward lower frequencies as the dose decreased (Table 1). The maximum amplitude of the TTF peak also decreased, particularly at low dose levels, for both kernels. In comparison with ASIR-V0, DLIR exhibited a better spatial resolution, especially at low dose for the Lung kernel. The longitudinal spatial resolution was very similar across dose levels, kernels, and algorithms, showing that kernels and algorithms do not influence longitudinal resolution (Fig. 3).

**Table 1:** Frequency of the TTF peak (in mm^-1^) for the lumbar spine protocol (kernel Bone+) and the chest protocol (kernel Lung) at three dose levels with five reconstruction algorithms, ASIR-V0, ASIR-V80, DLIR-Low, DLIR-Medium, and DLIR-High

	Lung			Bone+		
	0.8 mGy	3.0 mGy	7.0 mGy	3.0 mGy	7.0 mGy	15 mGy
AV0	0.286	0.386	0.386	0.501	0.501	0.441
AV80	0.225	0.235	0.245	0.245	0.185	0.000
DLL	0.301	0.366	0.361	0.531	0.546	0.506
DLM	0.326	0.381	0.361	0.521	0.541	0.506
DLH	0.316	0.376	0.381	0.501	0.541	0.506

*AV0* ASIR-V0, *AV80* ASIR-V80, *DLL* DLIR-Low, *DLM* DLIR-Medium, *DLH* DLIR-High

### Noise assessment

**Fig. 4:**
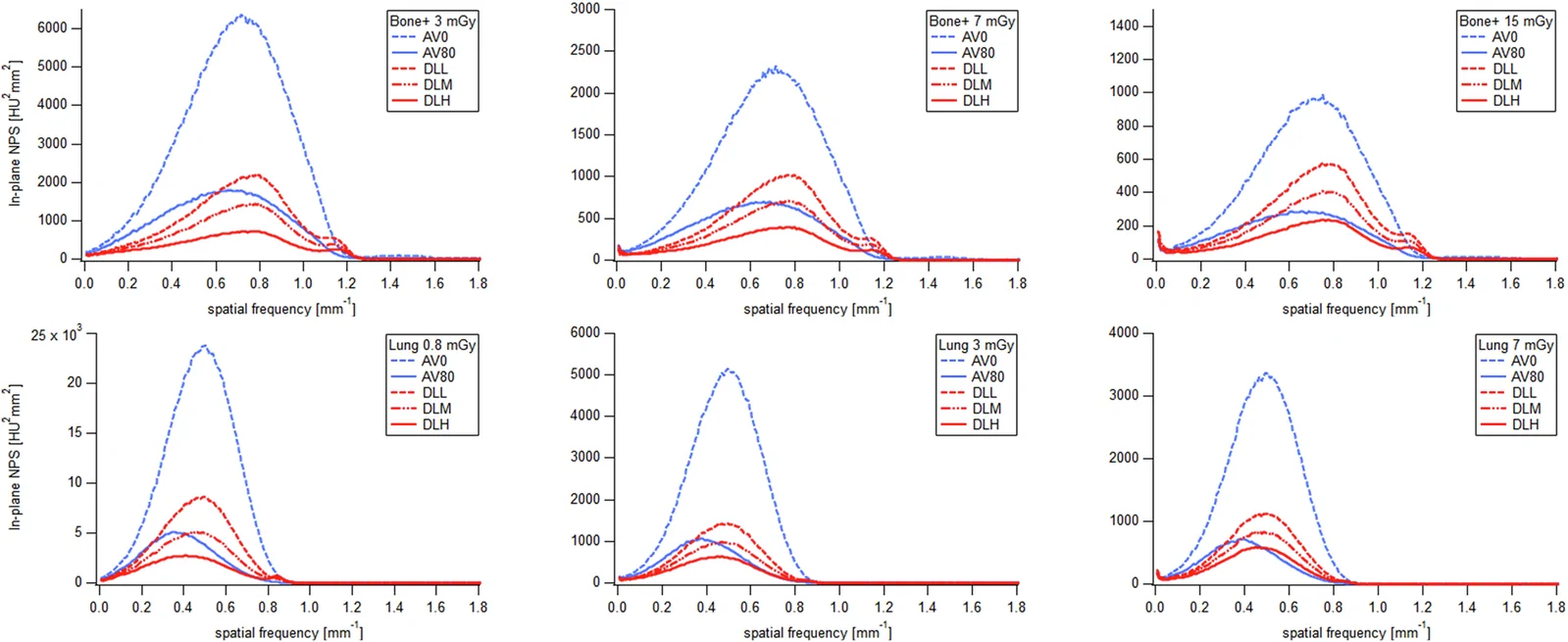
Noise power spectrum (NPS) for different dose levels/kernels for the five algorithms, ASIR-V0 (AV0), ASIR-V80 (AV80), DLIR-Low (DLL), DLIR-Medium (DLM), and DLIR-High (DLH)

Noise power spectra (NPS) were markedly influenced by the choice of the reconstruction algorithm. The noise level decreased with the dose and was higher for the Lung kernel than the Bone+ kernel (Fig. 4). ASIR-V0 consistently exhibited the highest noise magnitude. DLIR markedly reduced the image noise by factors between 1.9 and 2.8 for the Lung kernel and between 2.0 and 2.8 for the Bone+ kernel (Table 2).

**Table 2:** Average frequency of the NPS and noise magnitude ratio between ASIR-V0 and other algorithms, ASIR-V80, DLIR-Low, DLIR-Medium, DLIR-High, for the lumbar spine protocol (kernel Bone+) and the chest protocol (kernel Lung) at three dose levels

		Lung			Bone+		
		0.8 mGy	3.0 mGy	7.0 mGy	3.0 mGy	7.0 mGy	15 mGy
Average frequency of the NPS (f_av_, mm^-1^)	AV0	0.474	0.480	0.478	0.681	0.678	0.678
	AV80	0.382	0.390	0.389	0.622	0.616	0.613
	DLL	0.455	0.459	0.463	0.690	0.698	0.696
	DLM	0.441	0.447	0.455	0.679	0.685	0.683
	DLH	0.423	0.433	0.445	0.659	0.660	0.670
Noise magnitude ratio in comparison with AV0	AV0	-	-	-	-	-	-
	AV80	2.360	2.378	2.365	1.811	1.753	1.772
	DLL	1.667	1.900	1.732	1.708	1.508	1.311
	DLM	2.131	2.276	2.011	2.082	1.795	1.548
	DLH	2.852	2.818	1.963	2.825	2.343	1.994

*AV0* ASIR-V0, *AV80* ASIR-V80, *DLL* DLIR-Low, *DLM* DLIR-Medium, *DLH* DLIR-High

The noise magnitude ratio increased with the DLIR strength, decreased with the dose, and was higher for the Lung kernel. Only DLH gave lower noise than AV80 for all kernels and dose levels.

Reconstruction kernels and algorithms significantly influenced noise texture. The Bone+ kernel exhibited higher mean NPS frequencies than the Lung kernel, indicating a finer noise texture. With AV0 reconstruction, f_av_ ranged from 0.474–0.480 mm⁻¹ for the Lung kernel and 0.678–0.681 mm⁻¹ for the Bone+ kernel. AV80 reconstruction shifted the NPS toward lower spatial frequencies, reducing f_av_ to 0.382–0.390 mm⁻¹ for the Lung kernel and 0.613–0.622 mm⁻¹ for the Bone+ kernel, consistent with smoother noise texture. In contrast, DLIR reconstructions preserved a noise texture closer to AV0, with mean frequencies ranging from 0.423–0.463 mm⁻¹ for the Lung kernel and 0.659–0.698 mm⁻¹ for the Bone+ kernel, depending on the DLIR strength level (Table 2). At low dose levels, all algorithms showed a slight shift of the NPS peak toward lower spatial frequencies.

### Contrast

**Fig. 5:**
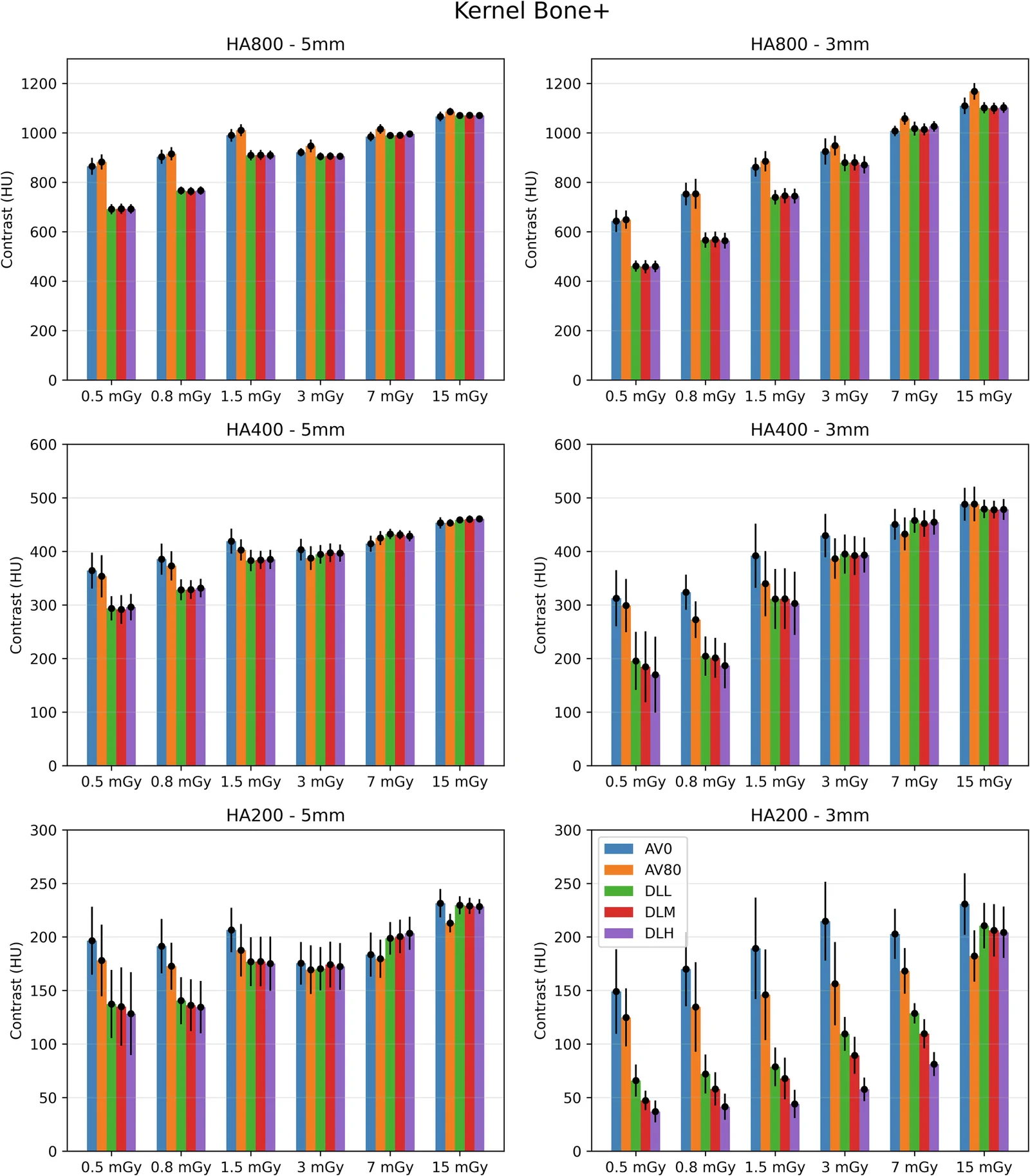
Contrast values of the six lesions (two lesion sizes, three concentrations) for the lumbar spine protocol (Bone+ kernel) and five reconstruction algorithms: ASIR-V0 (AV0), ASIR-V80 (AV80), DLIR-Low (DLL), DLIR-Medium (DLM), and DLIR-High (DLH). Error bars indicate the 95% confidence interval

For the lumbar spine protocol (kernel Bone+), the contrast for the six lesions tended to decrease at low dose for all reconstruction methods (Fig. 5). AV0 and AV80 consistently yielded a contrast closer to the baseline value, while DLIR systematically showed a contrast loss, more important for DLH than for DLL and DLM. The contrast loss worsened when the dose, lesion size, and concentration decreased.

**Fig. 6:**
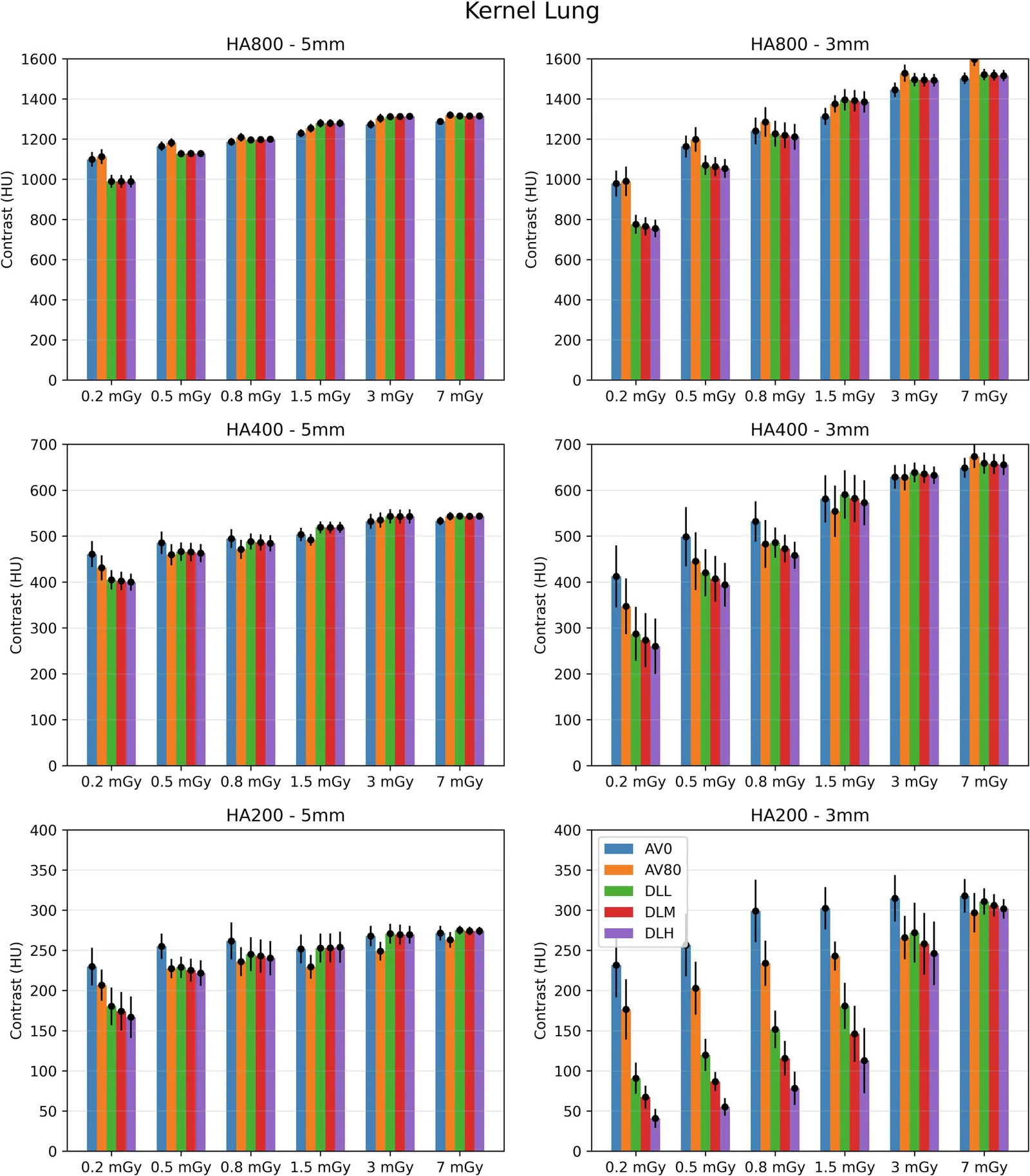
Contrast values of the six lesions (two lesion sizes, three concentrations) for the chest protocol (Lung kernel) and five reconstruction algorithms: ASIR-V0 (AV0), ASIR-V80 (AV80), DLIR-Low (DLL), DLIR-Medium (DLM), and DLIR-High (DLH). Error bars indicate the 95% confidence interval

At 0.5 mGy, DLH reduced contrast by up to 70% for the small low-concentration lesion (HA200, 3 mm), compared with 19% for the larger high-concentration lesion (HA800, 5 mm). In comparison, the contrast reduction was limited to a maximum of 11% for the highest dose level (15 mGy) across the different lesions. A similar trend was observed for the chest protocol (kernel Lung) (Fig. 6). ASIR-V consistently preserved contrast better than DLIR across all dose levels and lesion types. The contrast reduction with DLH reached 82% at 0.2 mGy for the lesion HA200/3 mm, and only 10% for the lesion HA800/5 mm, confirming that the contrast penalty is most severe for small, low-concentration lesions at very low dose. At the highest dose level, the contrast decreased by a maximum of 5% compared with ASIR-V0 across the different lesions.

### Detectability indices

**Fig. 7:**
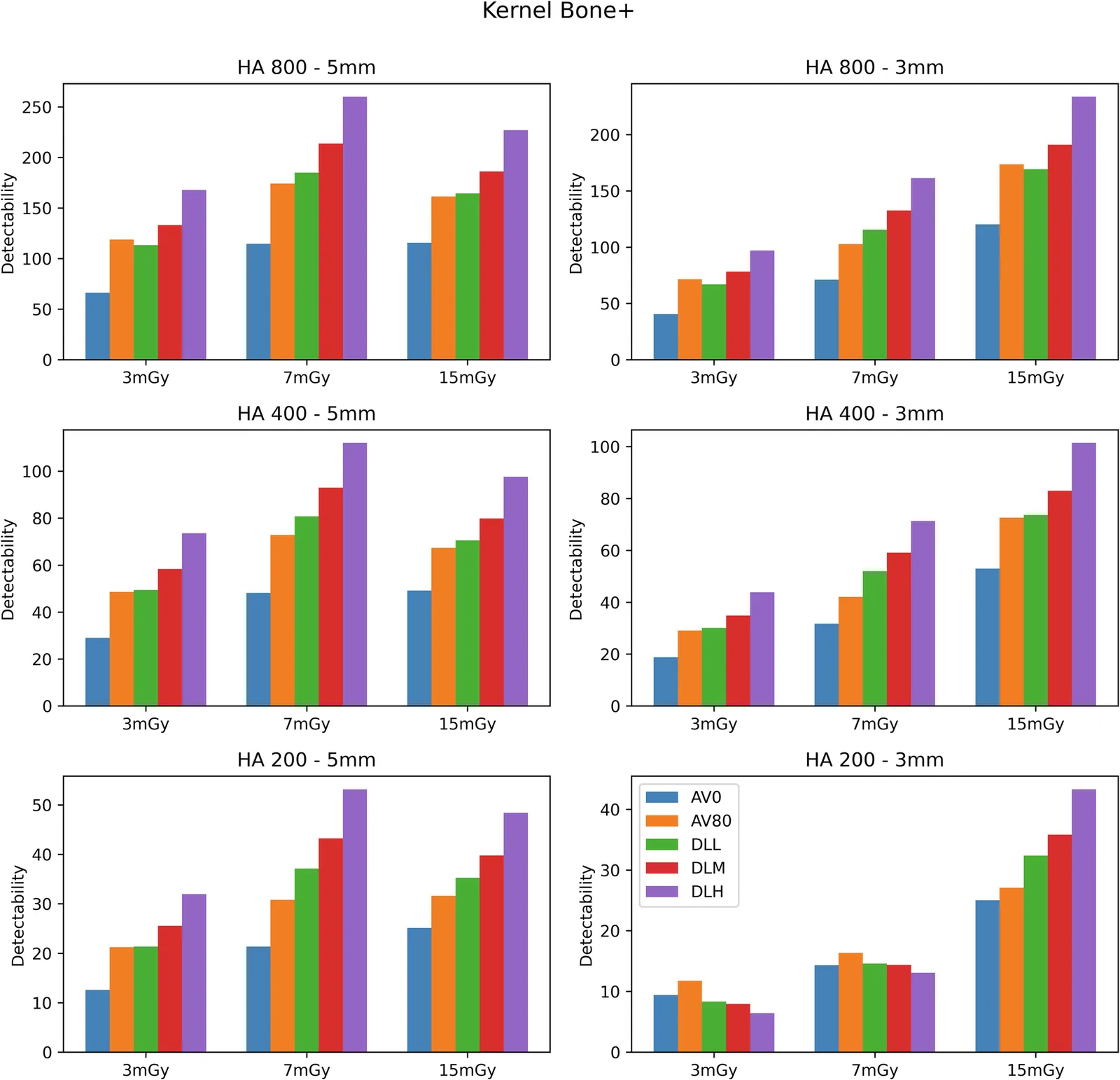
Detectability of the six lesions (two lesion sizes, three concentrations) for the lumbar spine protocol and five reconstruction algorithms: ASIR-V0 (AV0), ASIR-V80 (AV80), DLIR-Low (DLL), DLIR-Medium (DLM), and DLIR-High (DLH)

**Fig. 8:**
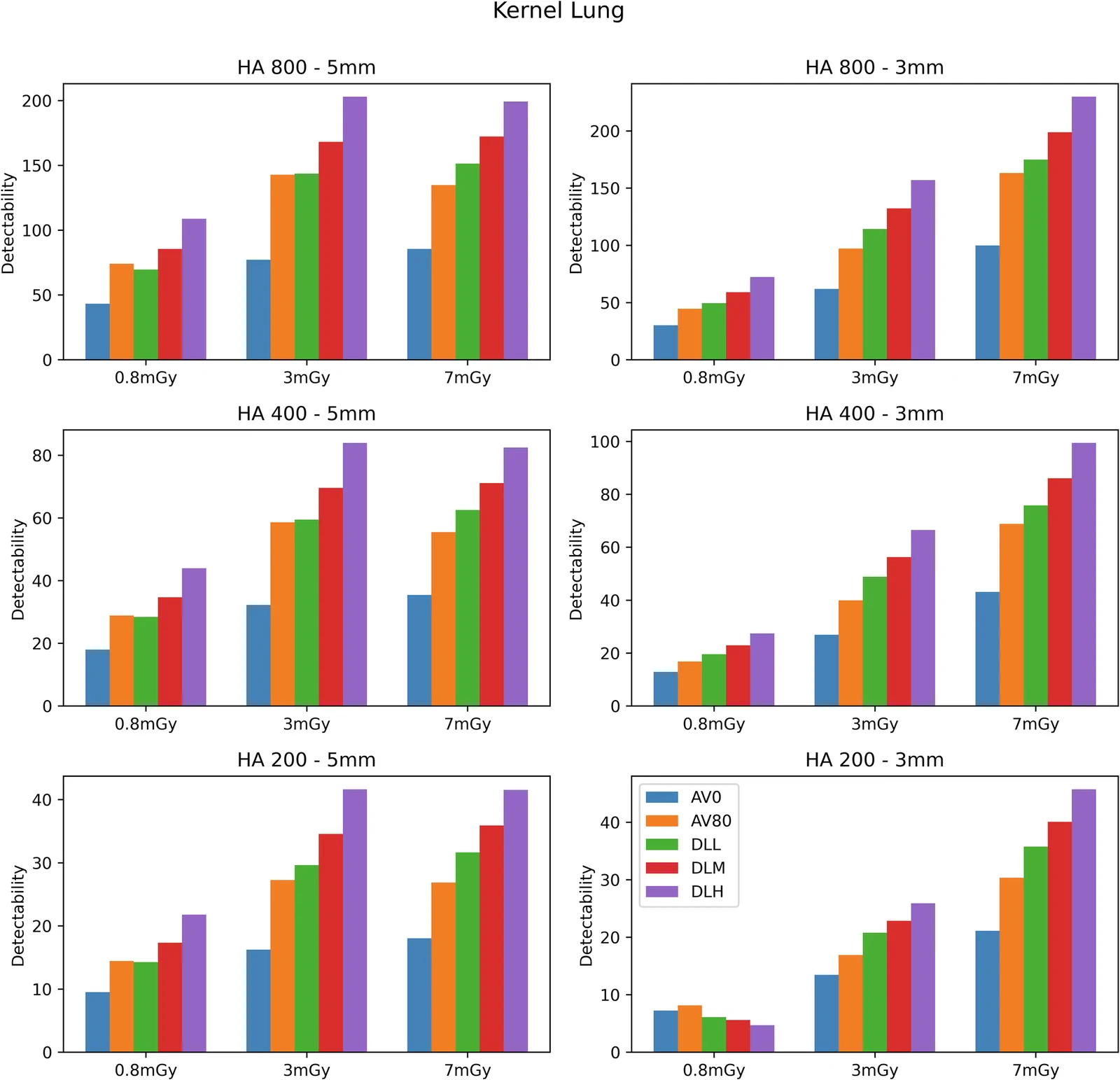
Detectability of the six lesions (two lesion sizes, three concentrations) for the chest protocol (Lung kernel) and five reconstruction algorithms: ASIR-V0 (AV0), ASIR-V80 (AV80), DLIR-Low (DLL), DLIR-Medium (DLM), and DLIR-High (DLH)

For the lumbar spine protocol with the Bone+ kernel (Fig. 7), increasing DLIR strength improved detectability, and DLH exhibited the highest detectability for all lesion sizes and concentrations at the highest dose of 15 mGy. DLIR equalized or outperformed ASIR-V in most situations, except for the smallest lesion size and concentration (HA200, 3 mm) at 3 and 7 mGy, where AV80 yielded the highest detectability. In these two cases, DLH exhibited a detectability value 45% lower than AV80 at 3 mGy and 20% at 7 mGy. A similar trend was observed for the chest protocol with the kernel Lung (Fig. 8). DLH provided the highest detectability at 3 and 7 mGy for all six lesions (resp. 53% and 51% higher than AV80). At ultra-low dose level (0.8 mGy), DLH exhibited a detectability 42% lower than AV80 for the smallest lesion size and the lowest concentration (HA200, 3 mm).

## Discussion

Recent CT evolutions have enabled the combination of high-frequency reconstruction kernels (like Bone + and Lung kernels) with deep-learning algorithm DLIR [20]. This study compared the performance of DLIR to conventional IR for two high-resolution CT protocols, a lumbar spine protocol with a high-definition kernel (“Bone+”) and a chest protocol with an edge-enhancing kernel (“Lung”). Contrast, spatial resolution, noise, and lesion detectability were influenced by the reconstruction algorithm and the kernel, with distinct trends observed between low and routine dose levels.

Compared to IR, DLIR maintained or improved the high-resolution features of CT images across all strengths in both protocols. While IR algorithms were dependent on both dose levels and lesion contrast, DLH showed a better spatial resolution at low dose levels. These results highlight that DLIR preserves spatial resolution more consistently than high-strength ASIR-V, which may be particularly relevant for detecting small or subtle structures as intervertebral disc calcifications or subsolid lung nodules. The performance of DLH applied to soft-tissue kernels was already described, and similar outcomes were obtained [14, 15]. Longitudinal resolution (TTF_z_) remained unaffected by either algorithm or kernel. The reconstruction algorithms only influence in-plane resolution, while TTF_z_ primarily depends on slice thickness and increment reconstruction [21].

Concerning noise, DLH consistently achieved higher noise reduction than AV80. In comparison with AV0, the noise was reduced by 90% with DLH and 70% with AV80. Notably, AV80 shifted the NPS toward lower frequencies compared with AV0 (equivalent to Filtered Back Projection), while DLIR preserved the noise frequency content of the original unprocessed images (AV0). This preservation of noise texture is clinically meaningful: the blotchy, low-frequency appearance introduced by high-strength IR algorithms such as AV80 is known to reduce radiologist confidence and alter lesion appearance [9, 10, 26]. By maintaining a natural grain-like texture while substantially reducing noise magnitude, DLIR overcomes this key limitation of IR [9, 10, 28]. These results are consistent with numerous studies demonstrating the potential of DLIR applied to soft-tissue kernels to reduce image noise [16–18, 28–30]. Racine et al [14] reported an 84% noise reduction with DLH compared to AV0 in a phantom study using a Standard kernel, and also observed a preservation of the image frequency content. Similar outcomes have also been reported with deep-learning reconstruction algorithms and high-frequency kernels implemented on different CT systems from other manufacturers [31, 32].

ASIR-V algorithms yielded better lesion contrast than DLIR for small, low-concentration lesions at low dose levels, while contrast differences were negligible at standard dose levels. Specifically, the contrast loss with DLH reached 70% for Bone+ and 82% for Lung kernel at the lowest dose level. This contrast reduction could be attributable to the behavior of DLIR in low-CNR conditions: when the signal-to-noise ratio is insufficient, the algorithm cannot reliably distinguish the signal of small structures from background noise and suppresses both indiscriminately, resulting in over-smoothing of low-contrast features. However, at the highest dose (15 mGy for the lumbar spine protocol and 7 mGy for the chest protocol), contrast differences between DLIR and ASIR-V were small (< 10%), suggesting that DLIR can be safely used without a substantial loss of contrast at standard dose levels. One strength of our study is the assessment of contrast for clinically relevant structures (in terms of contrast and size). This parameter has not yet been studied or has only been calculated on large inserts (such as those used for TTF calculation), which is not representative of clinical tasks [33, 34]. The loss of contrast observed for small and low-contrast lesions, not associated with a loss of spatial resolution, could explain the decrease in lesion conspicuity sometimes described for DLH and soft-tissue kernels [35–37].

Finally, DLH outperformed IR in detectability for most lesion sizes and concentrations. AV80 outperformed DLIR only for small and low-contrast lesions below 7 mGy for the Bone+ kernel and 3 mGy for the Lung kernel. Indeed, DLIR substantially reduces image noise and improves contrast-to-noise ratio (CNR), but high-strength settings (DLH) can reduce lesion conspicuity or “wash out” subtle, low-contrast lesions. Excessive smoothing in low-CNR scenarios can diminish subtle pathology detection compared to IR or moderate settings. This drawback could be critical as low-dose protocols are encouraged in chest imaging, especially in lung cancer screening, where dose levels lower than 1.5 mGy are recommended to minimize cumulative radiation exposure from repeated long-term screening rounds [38]. In this context, IR still offers advantages for detecting subtle lesions in low-dose scenarios while DLIR improves detectability performance in other cases, with potential for radiation dose reduction [28, 39, 40]. These findings emphasize that the optimal reconstruction strategy depend to both dose level and clinical task, balancing noise suppression, spatial resolution, and contrast preservation. Clinicians should therefore consider both the dose level and the specific diagnostic task when selecting reconstruction parameters, favoring moderate DLIR strengths (DLL or DLM) or iterative reconstructions in low-dose protocols targeting small or subtle lesions.

Although performed in a phantom setting, our study provides an important foundation for understanding the potential clinical applicability of DLIR techniques in lung and bone imaging and may help guide the selection of reconstruction settings and kernels according to the clinical indication, particularly in scenarios where low doses are recommended, for lung cancer screening or for evaluating pulmonary nodules or for assessing infectious pneumonias [38, 41]. Since the investigated dose levels were selected to reflect contemporary standard and low-dose CT practice, our findings provide a technical basis for the future clinical implementation and optimization of DLIR-based protocols.

Nevertheless, these results will still need to be confirmed by clinical studies where detection is performed on structured anatomical noise. The homogenous phantoms used in this study cannot reproduce the complexity of human anatomy, tissue heterogeneity, and motion effects encountered in clinical imaging. Detectability and image quality of CT images reconstructed using non-linear algorithms may be sensitive to the specific insert materials, lesion sizes, and contrast levels used in the phantom. In addition, the standardized phantom size cannot reflect the variability in patient body habitus encountered in routine clinical practice, which can significantly influence noise and image quality. Finally, although quantitative metrics provide objective comparisons between iterative and deep-learning algorithms, specific analysis on patient images should be conducted to confirm these findings. This study was conducted using a single scanner from one manufacturer. The specific algorithms used for high-resolution protocols proposed by the other manufacturers should be further validated through both quantitative and qualitative assessments.

In conclusion, DLIR combined with high-frequency kernels preserved spatial resolution and maintained a natural, FBP-like noise texture while substantially reducing image noise. These combined benefits translated into superior lesion detectability compared with IR for standard and high dose levels. However, at low dose levels, the aggressive noise suppression of DLH resulted in over-smoothing small, low-contrast structures, reducing their contrast and ultimately their detectability compared with IR. This contrast loss was dose-dependent and specific to small, low-concentration lesions in low-CNR conditions, and did not affect larger or higher-contrast structures. Therefore, while DLIR-High could be recommended for high-resolution CT protocols at standard dose levels, moderate DLIR strengths or iterative algorithms should be preferred in low dose acquisitions targeting small or subtle lesions, such as lung cancer screening, to avoid the trade-off between noise reduction and contrast preservation. The clinical relevance of these findings should be confirmed through human reader studies and clinical evaluations to assess diagnostic performance.

## Data Availability

Data will be made available to the reader on reasonable request.

## Abbreviations

AV0: ASIR-V0
AV80: ASIR-V80
CNR: Contrast-to-noise ratio
CT: Computed tomography
DLIR: Deep-learning iterative reconstruction
DLH: DLIR High
DLL: DLIR Low
DLM: DLIR Medium
FBP: Filtered back projection
IR: Iterative reconstruction
NPS: Noise power spectrum
NPWE: Non-prewhitening model observer with eye filter
PMMA: Polymethyl methacrylate
ROI: Region of interest
TTF: Target transfer function
VTF: Visual transfer function
